# Dissemination of a facilitation strategy to de-implement unnecessary post-operative antibiotics at children's hospitals: The Optimizing Perioperative Antibiotic in Children (OPerAtiC) trial 2.0

**DOI:** 10.1186/s13012-025-01460-5

**Published:** 2025-11-10

**Authors:** Emmanuel K. Tetteh, Harry Obeng, Andrew Atkinson, Sara Malone, Matt Sattler, Tyler Walsh, Lauren Walsh, Jacqueline M. Saito, Shawn J. Rangel, Jason G. Newland, Virginia R. McKay

**Affiliations:** 1 School of Public Health, Washington University in St. Louis, St. Louis, MO USA; 2 Division of Infectious Diseases, Department of Medicine, Washington University School of Medicine, St. Louis, MO USA; 3 Institute of Informatics, Data Science, and Biostatistics, Department of Medicine, Washington University in St. Louis School of Medicine, St. Louis, MO USA; 4 School of Public Health, Washington University in St. Louis, St. Louis, MO USA; 5 Division of Pediatric Infectious Diseases, Department of Pediatrics, Washington University in St. Louis School of Medicine, St. Louis, MO USA; 6 Center For Child Health Equity Outcome Research, Nationwide Children’s Hospital, Columbus, OH USA; 7 Division of General and Thoracic Surgery, Children’s National Hospital, Washington, DC USA; 8 Department of Surgery, Boston Children’s Hospital, Harvard Medical School, Boston, MA USA; 9 Division of Infectious Diseases, Nationwide Children’s Hospital, The Ohio State University College of Medicine, 700 Childrens Drive, Columbus, OH 43205 USA

**Keywords:** Antimicrobial stewardship, De-implementation, Facilitation, Implementation science, Dissemination strategies, Order set modification, Antibiotic overuse, Multi-center study

## Abstract

**Background:**

Excessive postoperative antibiotic use in pediatric surgical patients contributes to antibiotic resistance and increases the risk of *Clostridioides difficile* infection. Despite established guidelines recommending limited postoperative antibiotic duration, many hospitals struggle with de-implementation. This study aims to disseminate and evaluate the impact of a combined strategy to reduce unnecessary postoperative antibiotic use which combines enhanced antimicrobial stewardship program facilitation, defined as a set of actions to enable implementation, combined with order set review and modification. This multi-center study builds on an initial stepped wedge cluster randomized trial involving nine children’s hospitals, where facilitation training improved implementation of surgical prophylaxis guidelines and improved post-operative antibiotic use.

**Methods:**

The current study expands the strategy to a diverse set of hospitals caring for children in the US. Antimicrobial stewardship teams and surgeons from any hospital providing pediatric surgical care will be eligible to participate in facilitation training either as a single session webinar or as asynchronous modules. Based on the integrated Promoting Action on Research Implementation in Health Services (iPARiHS), the facilitation training includes didactic presentations and activities that focus on current evidence related to surgical prophylaxis, evaluation of context, interpersonal relationships, and structured processes to foster change. We will use a quasi-experimental time-series design collecting data from clinicians using a structured interview guide every six months on implementation of prophylaxis guideline congruent order sets as the primary implementation outcome. We will also evaluate trends in at least 20 hospitals collecting quality improvement data through the National Surgical Quality Improvement Program-Pediatrics (NSQIP-P) on postoperative antibiotic use, surgical site infections, and *Clostridioides difficile* infections from 2022–2027.

**Discussion:**

By scaling up this intervention, the study aims to provide a robust evaluation of its effectiveness across diverse hospital settings. If successful, this approach could inform future antimicrobial stewardship efforts in pediatric and adult surgical populations, offering a scalable model for reducing inappropriate antibiotic use while maintaining patient safety.

**Supplementary Information:**

The online version contains supplementary material available at 10.1186/s13012-025-01460-5.

Contributions to the literature

- This study advances de-implementation research by evaluating facilitation combined with order set modifications as a scalable strategy to reduce unnecessary postoperative antibiotic use in pediatric surgery, addressing a critical gap in antimicrobial stewardship.
- Unlike traditional antimicrobial stewardship interventions, this approach emphasizes theory-based interdisciplinary facilitation training to strengthen collaboration between stewardship teams and surgical departments.
- With a multi-center study design spanning more than 20 hospitals, this study enhances the generalizability of de-implementation strategies and evaluates long-term outcomes using time-series analysis.
- The study contributes to ‘dissemination science’ by examining how asynchronous training and targeted facilitation can support the spread and adoption of evidence-based prescribing practices.

## Background

Antimicrobial-resistant (AMR) infections, driven by excessive and inappropriate antibiotic use, are an increasing public health emergency. A dramatic increase in the carriage of multidrug-resistant organisms has been observed since 2000 with projections indicating that if no efforts are made to improve antimicrobial use, AMR infections could result in 10 million deaths annually by 2050, surpassing the annual number of deaths due to cancer (8 million) [1–4]. *Clostridioides difficile* infection (CDI) is an adverse event associated with antibiotic use and is a more direct and immediate harmful outcome. Hospitalized children who develop CDI face an increased risk of death, prolonged hospitalization by up to seven days, and additional hospital costs of approximately $100,000 [5].

Even a single dose of perioperative antibiotic prophylaxis increases the risk six-fold for CDI [6, 7]. Studies indicate that inappropriate pre- and post-operative antibiotic prophylaxis occurs in up to 56% of cases, with unnecessary postoperative doses administered in 55% of low-risk procedures (clean and clean-contaminated cases) [6, 8, 9]. Improving the use of surgical antibiotic prophylaxis in the postoperative period across children’s hospitals presents a significant opportunity to reduce inappropriate antibiotic overuse. In published guidelines from the Centers for Disease Control and Prevention and the American Academy of Pediatrics Committee on Infectious Diseases, both strongly recommend limiting surgical antibiotic prophylaxis to a single perioperative dose for clean and clean-contaminated cases [10]. Furthermore, they advise discontinuing prophylaxis once the surgical incision is closed for uncomplicated procedures [11].

Antimicrobial stewardship programs (ASPs), typically co-led by pharmacists and infectious diseases physicians, are a condition of participation by the Centers for Medicare & Medicaid Services. These programs play a crucial role in optimizing antimicrobial selection and duration, reducing the development of AMR, and improving patient safety. ASP team members are well-positioned to integrate evidence-based guidelines, as they collaborate with various clinical services (e.g., surgeons, hospitalists) to ensure appropriate antimicrobial selection, dosing, and duration for both treatment and prophylaxis. However, reducing unnecessary antibiotic use among surgeons remains challenging due to multiple barriers, including skepticism toward evidence, a desire to balance risk of surgical site infection (SSI) with the risk of excessive antibiotic use, entrenched prescribing habits, and limited rapport with ASP teams [12]. Bridging this gap is essential to fostering collaboration, enhancing guideline adherence, and ultimately improving the judicious use of antimicrobials in surgical care [13].

Facilitation is an evidence-based strategy that addresses contextual barriers to change, and allows for the communication of evidence effectively, and builds relationships with end users. This approach has been successful in bridging the relational gap and fostering collaboration between ASP teams and surgeons [14–16]. Our recent study (Operatic 1.0), using a stepped-wedge cluster randomized research design across nine U.S. children’s hospitals, trialed a baseline training with ASP teams focused on reviewing and modifying surgical order sets alone compared to order set modification supplemented with a facilitation training. Our results demonstrated the benefit of ASP teams attending a facilitation workshop to support modifications to electronic order sets over and above order set training alone, ultimately reducing unnecessary postoperative antibiotic use [16–18]. Expanding this combined strategy to a larger network of hospitals and ASP teams has the potential to further improve antibiotic prescribing practices for pediatric surgical patients and contribute to the growing evidence on the effectiveness of facilitation in de-implementing unnecessary healthcare practices.

The overall goal of the proposed study is to widely disseminate our facilitation workshop as a strategy to de-implement unnecessary post-operative antibiotics in children. To achieve this, we will: (1) update and adapt our facilitation workshop to make it accessible to over 150 children's hospitals in the United States, (2) evaluate implementation outcomes related to the workshop, assessing how the skills and knowledge gained impact the adoption of prescribing guidelines, reach through order set changes, and the application of facilitation techniques, and (3) evaluate the clinical impacts, including reductions in inappropriate antibiotic use and potential effects on surgical site infections and CDI across 20 hospitals. By the end of the dissemination project, we conservatively estimate that our efforts will impact approximately 50,000 surgical cases during the project period.

## Methods

### Study design

This study will use a quasi-experimental design with an interrupted time-series analysis to compare three years pre (2022–2024) and post (2025–2027) intervention. The intervention will involve training ASP teams and surgeons in facilitation strategies in addition to conducting order set review and modification. We hypothesize that a combined order set and facilitation training will result in a significant reduction in excessive post operative prophylaxis use, by fostering better engagement between ASP teams and surgeons, such as helping negotiate different communication styles and understanding of relevant data presentation. The primary endpoint of the study is the percentage of inappropriate antibiotic use. Secondary endpoints will investigate change in SSI and CDI rates.

The Nationwide Children’s Hospital Institutional Review Board (IRB) has deemed this study non-human subjects research (IRB #: STUDY00004505). An information sheet will be provided to adult ASP team members and surgeons completing the surveys. The Standard Protocol Items: Recommendations for Interventional Trials (SPIRIT) Checklist guided the development of this protocol manuscript [19].

### Study setting and participants

This study will be conducted at a minimum of 20 children's hospitals and will recruit from either members of the SHaring Antimicrobial Reports for Pediatric Stewardship (SHARPS) Collaborative [20, 21] or the National Surgical Quality Improvement Program-Pediatric (NSQIP-P) [22, 23]. Founded in 2013, SHARPS aims to improve antimicrobial use across healthcare settings and includes over 70 hospitals with dedicated antimicrobial stewardship teams [20]. NSQIP-P, with 156 participating hospitals, collects data on surgical procedures, including prophylactic antibiotic administration, to enhance pediatric surgical care [22]. ASP teams and surgeon champions from 20 hospitals will be recruited to participate in the updated facilitation workshop via live webinar or asynchronous online at least version. Recruitment will be facilitated through the SHARPS Collaborative Listserv and NSQIP-P webinars, which reach over 150 hospitals. Primary participants will be ASP team members, with extended eligibility for infectious diseases fellows, NSQIP-P surgical leads, surgical clinicians, and surgical fellows.

### Conceptual frameworks and logic model

Our study is guided by the i-PARIHS framework (Integrated Framework for Promoting Action on Research Implementation in Health Services), which facilitates the implementation of evidence-based practices [15, 24]. This framework, used in various clinical settings and informed by our previous Operatic 1.0 trial, posits that successful implementation depends on the quality of evidence, the context, and the facilitation process [15, 25, 26]. The four key constructs are: innovation (evidence quality), recipients (those affected by the evidence), context (the setting for implementation), and facilitation (the active process of integrating evidence into practice). For this study, we focus on contextual factors at both the hospital and surgical specialty levels, as these are where ASP activities are primarily implemented. A description of the variables characterizing the core constructs for this study is provided in Table 1. Successful evidence implementation requires facilitation strategies that assess and respond to both the evidence and recipients within their specific context. In our study, facilitation will be carried out by antimicrobial stewards who will use targeted strategies to enable the uptake and integration of evidence-based practices.

**Table 1:** Core i-PARIHS constructs

Innovation	Recipients	Context	Facilitation
1. Current antibiotic prophylaxis guidelines 2. Local prescribing & infection rates 3. Alignment of evidence with local priorities	1. Section Chiefs 2. Surgeons 3. Patients 4. Supportive staff	1. Resources 2. Culture 3. Evaluation Receptivity 4. Leadership	1. Antimicrobial Stewards 2. Order set review & modification 3. Recipient engagement

**Fig. 1:**
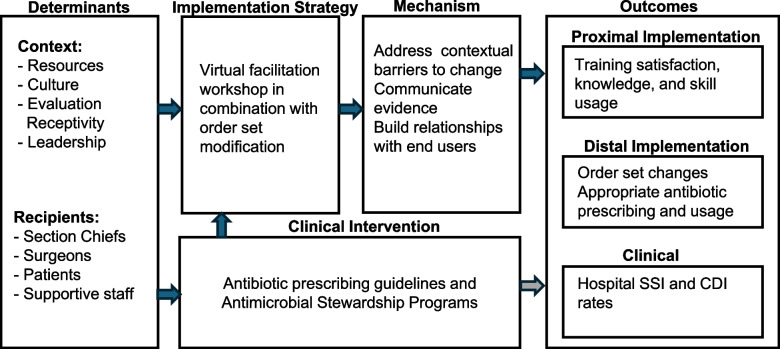
Implementation research logic model of proposed study

Figure 1 illustrates our implementation research logic model for improving antibiotic prescribing practices through a virtual facilitation workshop and order set modifications. The model highlights key contextual factors, including resources, culture, leadership, and receptivity, while identifying key actors such as surgical section chiefs, surgeons, and supportive staff. The intervention addresses barriers, promotes evidence-based practices, and fosters stakeholder engagement using antibiotic prescribing guidelines and ASP. Outcomes are categorized into proximal implementation outcomes (acceptability, satisfaction, and knowledge and skills acquisition), distal implementation outcomes (order set adoption and reach, appropriate antibiotic prescribing and usage), and clinical outcomes (SSI and C. diff rates).

### Facilitation workshop

#### Workshop updates and adaptation

In the first six months of the study, we will refine and consolidate our baseline order set and facilitation workshop to enhance scalability and accessibility. Participant feedback from our Operatic 1.0 trial has identified key improvements, including more team-based discussions, additional materials, reduced time commitment, and alignment with the latest evidence on de-implementation, facilitation, and surgical prophylaxis. To improve feasibility and dissemination, we will incorporate continuing medical education (CME) credits through Nationwide Children’s Hospital’s CME department, and offer a self-paced, asynchronous format alongside the live webinar. To address concerns about the workshop’s time demands (previously eight hours over four weeks), we aim to streamline it to three hours delivered in just one session instead of four. We will pre-record didactic content and seek permission to share interactive elements, such as role-playing exercises, ensuring a balance between structured learning and collaboration.

#### Workshop content and delivery

This workshop is designed to build facilitation skills in the context of de-implementation within ASPs. Drawing on real-world data and evidence-based strategies, the session guides participants through a structured exploration of behavior change, communication, and the use of local data to support reductions in post-operative surgical prophylaxis (Table 2). The live workshop combines didactic presentations, hands-on assessments, reflective exercises, and facilitated discussions. Participants will engage with core topics such as evidence-based prescribing practices, data-driven change strategies, hospital context and capacity, and interpersonal dynamics, including communication and conflict styles. Throughout, they will apply these skills to their own institutional settings, using tools like NSQIP-P reports and to identify opportunities for change.

**Table 2:** Facilitation training modules and content

Module	Content
De-implementation in ASP	• Overview of implementation science and the role of ASPs in change • Introduction to structured change strategies (e.g., order sets, facilitation) • Real-time reflection on current practices and data
Understanding the Evidence	• Review of current guidelines (e.g., WHO, SHARPS) • Analysis of NSQIP-P data and surgeon perspectives • Use of anecdotal evidence and local outcomes to drive change
Understanding Context	• Introduction to hospital context and relevant frameworks • Identification of site-specific barriers and opportunities • Structured activity to assess local capacity for improvement
Honing Facilitation Skills	• Assessment and reflection on communication and conflict styles • Exploration of team dynamics and facilitative leadership • Application of styles to support ASP initiatives
Presenting Evidence & Visualization	• Techniques for presenting data persuasively to clinical teams • Principles of effective evidence visualization • Guidance on pitching interventions and forming multidisciplinary teams
Wrap-Up and Next Steps	• Review of key takeaways • Guidance for applying workshop lessons at participants' institutions • Follow-up opportunities through collaborative meetings and study participation

Participants will also learn techniques for presenting and visualizing evidence to effectively influence surgical colleagues and promote team-driven, sustainable practice improvements. The live webinar will be offered quarterly over a two-year period, training approximately 20 participants per session. An asynchronous version, including recorded content, complementary materials, and interactive activities, will be available through the Nationwide Children’s Hospital CME website for ongoing access. Completion of the training provides access to quarterly collaborative meetings and the opportunity to contribute to ongoing data collection as part of the broader study.

Similarly, we will provide an asynchronous version of the workshop that will include pre-recorded presentations, activities, and opportunities to engage with Operatic community who have also participated in the workshop.

### Data collection

Assessing how facilitation supports the integration of surgical prophylaxis guidelines is key to measuring our workshop’s impact. To do so, we will track proximal implementation outcomes (acceptability, reach, and reaction) after training, as well as distal outcomes (e.g., order set changes, facilitation techniques) at 6, 12, and 18 months for all hospitals that participate in the training. For a minimum of 20 hospitals participating in NISQIP-P, we will evaluate key outcomes over time using a quasi-experimental time-series design, comparing three years pre- (2022–2024) and post-intervention (2025–2027). This approach balances internal validity with real-world feasibility and allows for detecting meaningful clinical effects.

#### Baseline data

##### Individual participant survey

Upon registration for the facilitation workshop, each participant will be invited to complete a survey that will collect data on their professional roles, experience, affiliation, and formal training in antimicrobial stewardship. Additionally, it will assess their engagement in stewardship decisions, and primary responsibilities related to antimicrobial use.

##### Hospital survey

Each participating hospital will be required to complete a characteristics survey, to be filled out by the ASP team member most familiar with the hospital. This survey will collect data on hospital size (e.g., number of beds, surgical volume) and details about the ASP program, including the strategies implemented, locations of implementation (e.g., intensive care units, emergency department), and evaluation metrics used. Additionally, the survey will gather specific information on ASP efforts to reduce post-operative antibiotic use in various surgical subspecialties.

#### Implementation data

##### Training feedback and evaluation survey

The Kirkpatrick framework will guide our assessment, evaluating training outcomes across four levels: Reaction (participant satisfaction), Learning (knowledge, attitude, and practice change), Behavior (application of learning), and Results (overall impact on performance or outcomes) [27]. Our post-training survey will assess the first two levels: Reaction and Learning. To assess participant satisfaction (Reaction), we will include ten items adapted from a previous assessment of workshop-based practice facilitation resources [16, 28]. To measure knowledge, and practice changes (Learning), participants will respond on a 5-point Likert scale, indicating their level of agreement or disagreement with statements assessing their knowledge, attitudes, and practices, related to facilitation strategies and antibiotic prophylaxis, before and after the workshop (see Supplementary File 1). The survey instrument was developed to align with the facilitation training modules and content (see Table 2). Data will be collected from workshop participants via a survey delivered through REDCap. A retrospective post-test survey will be used to assess both training and implementation outcomes [29, 30]. Participants will complete the survey upon finishing the workshop and will be asked to retrospectively evaluate their knowledge, attitude, and practices before the workshop and how these may have changed as a result of their participation. This approach helps mitigate potential biases in self-reporting that may arise with traditional pre-survey assessments and minimizes the under- or overestimation of initial knowledge [30]. It also simplifies the evaluation process, particularly when collecting pre-survey data is challenging [29].

##### Order sets review

To evaluate Behavior and Results domains of our evaluation model, an assessment will be conducted through 10-min structured interviews approximately 6-, 12-, and 18-months post-workshop training. An interview guide is provided in Supplementary File 2. Based on our experience from our Operatic 1.0 trial, this time interval is sufficient for ASPs to implement order set modifications across different surgical specialties, and participants are generally able to recall the work they have accomplished. The interview will inquire whether an order set modification was made, or a new order set developed (Yes/No, and number). For each impacted order set, we will inquire about the targeted procedure, the previous duration (no post-op antibiotics, 24 h or less, 25–48 h, or 48 h or more), and any changes in duration (eliminated, shortened, or no change). If the post-operative duration has been shortened, we will specify to what extent: no post-op antibiotics, 24 h or less, or 25–48 h. We anticipate some loss to follow-up due to staff turnover. In such cases, we will attempt to identify an alternative respondent, describe the study, and determine whether guideline integration through order set changes has continued.

#### Clinical data

Clinical data will be gathered using the NSQIP-P database to assess overall impact on appropriate post-operative antibiotics prescribing and usage. De-identified, case-level clinical outcome data from NSQIP-P will be requested from each hospital shortly after participation in the workshop and subsequently on an annual basis until we have obtained three years of data post workshop participation. Each participating hospital adheres to the protocols and definitions outlined in the NSQIP-P Manual of Operations. Data abstraction at each site is performed by a full-time NSQIP-P data abstractor, typically a pediatric nurse with clinical expertise, who undergoes thorough training to ensure precise and reliable data collection. Data are gathered for 35 cases every 8 days at each hospital, contributing to a dataset of up to 1,500 cases annually. Since December 2018, antibiotic prophylaxis data for each case includes the type of antibiotic used, the timing of administration (within 1 h before incision), whether redosing occurred, and the duration of postoperative antibiotic use. Data collection continues for 30 days post-surgery to monitor for potential complications, such as SSI and CDI, which may appear up to a month after the procedure. Data elements include basic demographics (age, sex, date of procedure), type of procedure, wound class (clean, clean-contaminated, contaminated, or dirty), comorbidities, and resources utilized during surgery.

## Statistical analysis plan

### Implementation outcomes

#### Proximal implementation outcomes

##### Facilitation workshop satisfaction and acceptability

Our analysis will address two key questions: (1) participant satisfaction with the mode and delivery of the facilitation workshop and (2) the perceived acceptability of the workshop. To assess these outcomes, we will summarize participant ratings on a five-point Likert scale (1 = strongly disagree, 2 = disagree, 3 = neutral, 4 = agree, 5 = strongly agree) using descriptive statistics (see Supplementary File 1). Open-ended survey responses will be analyzed using a thematic coding approach to identify key qualitative insights [31]. All responses will be systematically reviewed, and themes will be derived inductively by identifying and labeling meaningful units of text relevant to the research objectives.

##### Knowledge, attitude, and practices change

We hypothesize that participants' knowledge, attitude, and practices regarding the use of facilitation and order set modifications to improve antibiotic prescribing guideline adherence will improve from pre- to post-workshop training. First, to assess the reliability of the scale and subscale scores, we will use Cronbach’s alpha. Descriptive statistics and weighted means will be applied to compare participants’ reactions and learning outcomes pre- and post-workshop using Hedges and Olkin’s method [32]. Paired-sample t-tests will be conducted for each session to evaluate statistically significant differences between (suitably normalized) retrospective pretest and posttest scores.

#### Distal implementation outcomes (order set changes)

We will also analyze distal implementation outcome measures descriptively, using these results to inform analyses of clinical outcomes. In our Operatic 1.0 study, we encouraged participants to target surgical procedures that they felt were modifiable, and, as a result, different ASPs targeted different procedures in their respective hospitals. We will descriptively analyze these results to understand which procedures are successfully targeted for order set changes and which facilitation skills are being used over time. This information will guide our clinical analyses to understand what surgical specialties and/or specific procedures are likely to have been most impacted (e.g., spine procedures). As appropriate, we will fit general linear models to evaluate change in our implementation outcomes before and after the workshop long term.

### Clinical outcomes

#### Primary hypothesis

**Fig. 2:**
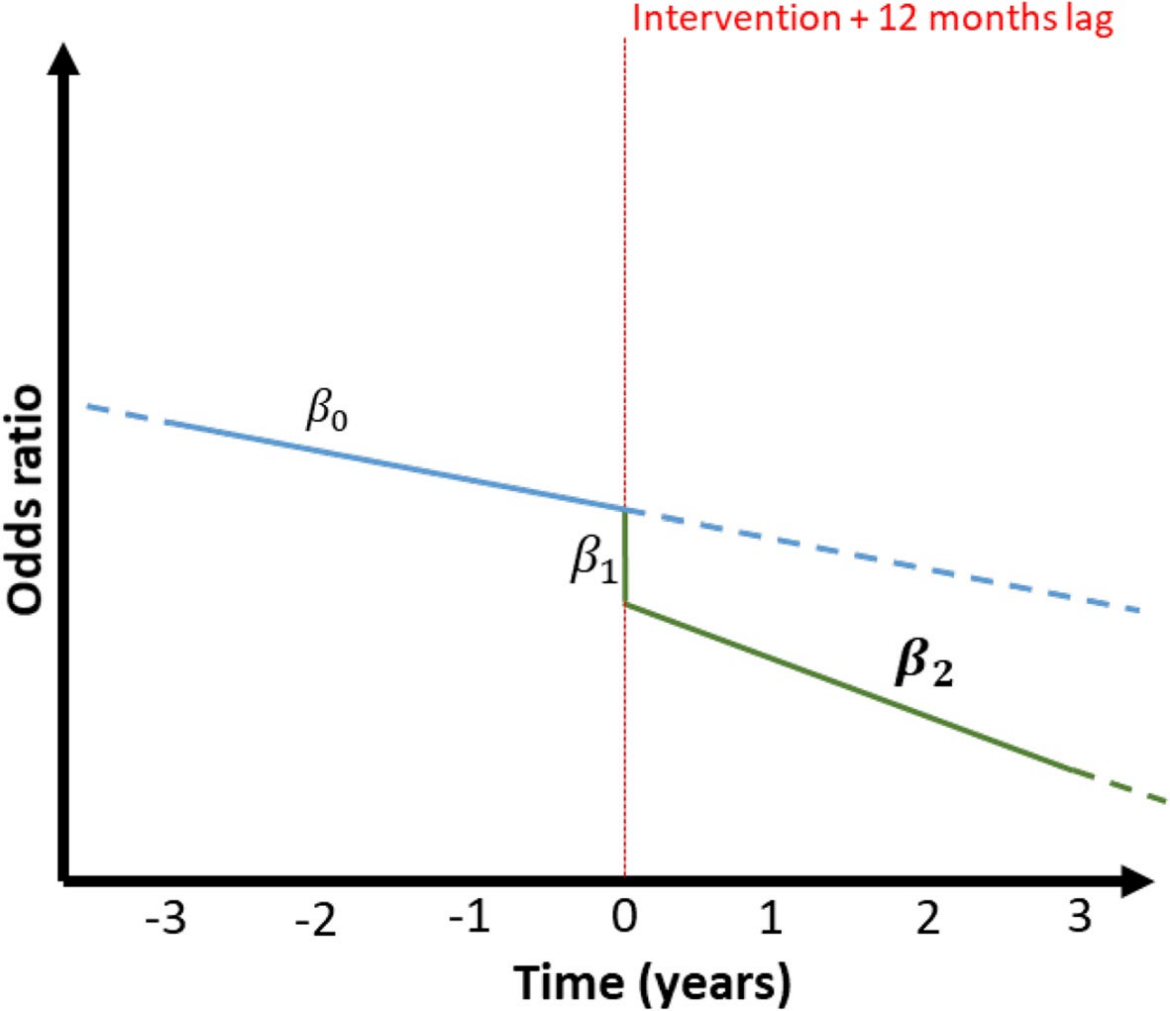
Illustrative example of the interrupted time series approach with estimated coefficients; ${\beta }_{0}$ the trend prior to the intervention, ${\beta }_{1}$ the immediate (local) effect following the intervention; ${\beta }_{2}$ the post-intervention trend (target population measure)

We hypothesize that the appropriately lagged post-intervention percentage of cases receiving unnecessary postoperative antibiotic prophylaxis will be significantly lower than in the pre-intervention period. To test this hypothesis, we will use an intention-to-treat approach for the primary analysis, including all complete case patient data from the NSQIP-P analysis set. A mixed-effects logistic interrupted time series (ITS) regression model will assess the impact of "order set change resulting in reduction" (1) versus "no order set change" (0) on prophylaxis prescriptions. The model will include the intervention (pre vs. post), time (in months), and an interaction term between time and intervention, including a random intercept to account for intra-hospital correlation in the model. The post-intervention slope (β_2) will be the primary estimate of interest (Fig. 2), but we will also investigate differences at 12, 24 and 36 months. The interruption point will be 12 months after each hospital’s first training, with sensitivity analyses at lags 9, 15, and 18 months as well. We will extend the model to adjust for age, surgical subspecialty, procedure type, hospitalization duration, and co-morbidities using Akaike information criterion (AIC)-based variable selection. Subgroup analyses by procedure type and hospital will also be conducted.

#### Secondary hypotheses

We will also test multiple secondary hypotheses to assess the additional impact of the implementation strategy. These include: (1) the rate of SSI does not change post-intervention and (2) the rate of CDI decreases post-intervention. Analogous ITS-type statistical models will be fitted to investigate secondary outcomes. All estimated effects will be reported as odds ratios with 95% confidence intervals and p-values. The tests will be two-sided with a 5% statistical significance level and will be conducted using R statistical software.

#### Missing data

Based on our previous studies, since we do not foresee a large percentage of either endpoint or covariate missingness in the data, the primary analysis will consider the complete case data only. If the level of missingness exceeds 5% for any specific covariate of interest, then multiple imputation under the missing at random (MAR) assumption will be performed [33]. The estimates from the fitted models with these full data sets will be compared with those from the complete case analysis, and any discrepancies will be discussed in subsequent scientific publications. Furthermore, following these analyses if the MAR assumption is deemed questionable, then appropriate sensitivity analyses will be performed to investigate plausible departures from this assumption [34].

#### Power and sample size calculations

The sample size calculation is based on the primary outcome of the percentage of cases in which unnecessary postoperative antibiotic prophylaxis is administered among clean and clean-contaminated cases in the 20 participating hospitals. For the purposes of the power calculation, the staggered temporal implementation of the intervention in each of the hospitals is approximated by assuming a stepped-wedge cluster randomized trial design and adopting the sample size formula and associated web-based tool of Hemming and colleagues [35]. Utilizing data from our previous clinical trial specific to spine procedures [36], this sample size calculation depends on the targeted effect size (a percentage decrease from 45% measured in the pre-intervention period), the number of steps (10, with two hospitals implementing the intervention approximately every month over a period of 12 months), the estimated number of procedures per month (14 per hospital per month), and the intra-cluster correlation (ICC) coefficient (estimated at 0.12 from previous studies) [37]. With 20 hospitals implementing the intervention over a (1 [pre-] + 1 year [training] + 3 [post] =) 5 year study period, we envisage attaining at least 80% power to detect a difference in the pre- and post-intervention outcome measure of at least 5%, with a (two-sided) statistical significance level of 5% (and no missing data). Here we assume that existing surveillance measures capture data for the pre-intervention period and make the conservative assumption that all participating hospitals have one or more years pre-intervention surveillance data (not part of the study period), and *at least* 3 years post-intervention follow-up, allowing for the 12-month lag in post-training effect.

## Discussion

This study aims to disseminate and evaluate the impact of enhanced antimicrobial stewardship program facilitation training, combined with order set review and modification, as de-implementation strategies to reduce postoperative antibiotic use in pediatric surgical patients. Our facilitation strategy proved effective in an initial trial involving nine children's hospitals in the United States as ASP teams successfully modified order sets, implemented surgical prophylaxis guidelines, and improved post-operative antibiotic use [16–18]. The current study will refine our strategy based on feedback and lessons learned from the earlier trial, scaling it up to more than 20 children's hospitals within the NSQIP-P and SHARPS Collaborative networks. If successful, our strategies will help reduce excessive antibiotic use, lower the incidence of CDI, and preserve the efficacy of antibiotic treatments for the future.

Our facilitation workshop introduces a novel approach to reducing inappropriate antibiotic use. Multiple systematic reviews of ASPs indicate that most hospital-based strategies for improving antibiotic use do not emphasize collaboration between ASPs and other specialties [38–40]. In contrast, antimicrobial stewardship professionals who participated in our training reported valuable benefits from the program’s focus on “soft skills”, such as communication techniques aimed at fostering collaboration and rapport [16]. Facilitation, as an implementation strategy, has been shown in other studies to support the development of these skills [14, 16, 41, 42]. By providing this workshop training to ASPs, surgeons, and other clinicians involved in reducing postoperative antibiotic use, we anticipate additional benefits, equipping participants with skills applicable across various specialties to de-implement unnecessary antimicrobial prescribing and use.

Although implementation science has gained significant traction, substantial gaps remain in the dissemination of evidence-based interventions and strategies [43–45]. Research on the mechanisms and outcomes of implementation strategies in healthcare has often not included dissemination strategies [46], resulting in a limited evidence base to guide effective dissemination efforts. Consequently, dissemination remains an underexplored area and is rarely evaluated through empirical studies [40, 44, 45]. The primary goal of our study is to widely disseminate our facilitation strategy to a broader audience to de-implement unnecessary post-operative antibiotic use at children's hospitals. We will use several approaches to achieve this. First, we will leverage established networks (SHARPS Collaborative and NSQIP-P) to expand the reach of our facilitation strategy. Second, we will engage former participants of our Operatic 1.0 training as facilitators, fostering a continuous exchange of knowledge and best practices. Third, we will incentivize participation by offering CME credits to workshop participants. Lastly, by incorporating both synchronous and asynchronous approaches, we aim to enhance accessibility and engagement across different locations.

A key strength of this study is its multi-center design, which enhances the generalizability of findings across diverse hospital settings. The inclusion of at least 20 hospitals participating in both the SHARPS Collaborative and NSQIP-P ensures that the intervention is tested in varied environments with different levels of ASP maturity and institutional support. Additionally, the use of a time-series analysis allows for the evaluation of long-term trends and the sustainability of the intervention’s effects. However, this study has potential limitations. The quasi-experimental design and interrupted time series analysis, while robust, do not entirely eliminate the possibility of confounding variables that may not have been captured or controlled for in our hospital demographics and individual participant characteristics, potentially influencing the observed outcomes. Additionally, reliance on NSQIP-P data for clinical outcomes may introduce delays in data availability. Another challenge is participant attrition, particularly in follow-up interviews assessing long-term implementation outcomes. While targeted recruitment and incentives will be used to mitigate this issue, some loss to follow-up is expected. Lastly, because this study is mostly conducted in tertiary-care children's hospitals, its findings may not be generalizable to other clinical settings, such as community hospitals.

The results of this study will inform future efforts to refine and optimize ASP facilitation strategies. If successful, the training model could be expanded to other pediatric and adult surgical settings, with potential adaptations based on specialty-specific prescribing patterns. This study represents a significant step toward improving antibiotic stewardship in pediatric surgery by addressing both the technical and relational aspects of implementation. The findings will provide critical evidence for disseminating and implementing facilitation and order set modifications to de-implement unnecessary post-operative antibiotics at children's hospitals in the United States.

## Supplementary Information

Supplementary Material 1.

Supplementary Material 2

Supplementary Material 3

## Data Availability

Not applicable.

## Abbreviations

AMR: Antimicrobial-resistance
ASP: Antibiotic Stewardship Program
CDI: Clostridioides difficile infection
CME: Continuing Medical Education
iPARIHS: Integrated Framework for Promoting Action on Research Implementation in Health Services
NSQIP-P: National Surgical Quality Improvement Project-Pediatrics
SHARPS: SHaring Antimicrobial Reports for Pediatric Stewardship
SSI: Surgical Site Infection
