# Supplementary material for: Dissemination of a facilitation strategy to de-implement unnecessary post-operative antibiotics at children's hospitals: The Optimizing Perioperative Antibiotic in Children (OPerAtiC) trial 2.0
Source: Implement Sci. 2025 Nov 10;20:49. doi: 10.1186/s13012-025-01460-5 (PMC12599022; doi:10.1186/s13012-025-01460-5)
Supplement: Supplementary file 1 — Supplementary Material 1. [file 13012_2025_1460_MOESM1_ESM.pdf]

Section 1. Workshop Evaluation		
**1.**	As a facilitator, would you consider yourself:	❑ Novice<br>❑ Experienced<br>❑ Expert<br>❑ I don't consider myself a facilitator
**2.**	How would you rate the workshop overall?	❑ Very Poor / Very Little<br>❑ Poor / Little<br>❑ Neutral / Moderate<br>❑ Good / Much<br>❑ Very Good / Very Much
**3.**	How valuable did you find the information on NSQIP data?	❑ Very Poor / Very Little<br>❑ Poor / Little<br>❑ Neutral / Moderate<br>❑ Good / Much<br>❑ Very Good / Very Much
**4.**	How helpful did you find the case studies in the communication and conflict session?	❑ Very Poor / Very Little<br>❑ Poor / Little<br>❑ Neutral / Moderate<br>❑ Good / Much<br>❑ Very Good / Very Much
**5.**	How helpful did you find the role play in the final session?	❑ Very Poor / Very Little<br>❑ Poor / Little<br>❑ Neutral / Moderate<br>❑ Good / Much<br>❑ Very Good / Very Much
**6.**	Do you think the workshop would be helpful in planning for implementation in other programs?	❑ Very Poor / Very Little<br>❑ Poor / Little<br>❑ Neutral / Moderate<br>❑ Good / Much<br>❑ Very Good / Very Much
**7.**	How helpful did you find the data visualization tips and tricks?	❑ Very Poor / Very Little<br>❑ Poor / Little<br>❑ Neutral / Moderate<br>❑ Good / Much<br>❑ Very Good / Very Much
**8.**	How useful did you find the session on communication styles?	❑ Very Poor / Very Little<br>❑ Poor / Little<br>❑ Neutral / Moderate<br>❑ Good / Much<br>❑ Very Good / Very Much
**9**	How helpful did you find the session on conflict styles?	❑ Very Poor / Very Little<br>❑ Poor / Little

		❑ Neutral / Moderate<br>❑ Good / Much<br>❑ Very Good / Very Much
**10**	As far as time spent, would you like the workshop to be	❑ Shorter<br>❑ The same length<br>❑ Longer

Section 2. Knowledge, Attitudes and Practices
The following questions will assess changes in your knowledge, attitudes, and practices before and after the workshop. Please indicate your level of agreement for each statement both before and after your participation in the workshop.

Know-ledge	Before the workshop	After the workshop
**11.**	I was familiar with the concept of de-implementation in the context of antimicrobial stewardship (ASP).<br>❑ Strongly Disagree<br>❑ Disagree<br>❑ Neutral<br>❑ Agree<br>❑ Strongly Agree	I am familiar with the concept of de-implementation in the context of antimicrobial stewardship (ASP)<br>❑ Strongly Disagree<br>❑ Disagree<br>❑ Neutral<br>❑ Agree<br>❑ Strongly Agree
**12.**	I understood the key principles of implementation science and how they apply to ASP<br>❑ Strongly Disagree<br>❑ Disagree<br>❑ Neutral<br>❑ Agree<br>❑ Strongly Agree	I understand the key principles of implementation science and how they apply to ASP<br>❑ Strongly Disagree<br>❑ Disagree<br>❑ Neutral<br>❑ Agree<br>❑ Strongly Agree
**13.**	I was knowledgeable about the evidence supporting the de-implementation of unnecessary post-operative antibiotics in pediatric settings.<br>❑ Strongly Disagree<br>❑ Disagree<br>❑ Neutral<br>❑ Agree<br>❑ Strongly Agree	I am knowledgeable about the evidence supporting the de-implementation of unnecessary post-operative antibiotics in pediatric settings.<br>❑ Strongly Disagree<br>❑ Disagree<br>❑ Neutral<br>❑ Agree<br>❑ Strongly Agree
**Attit-udes**	**Before the workshop**	**After the workshop**
**14.**	I believed unnecessary post-operative antibiotic use in children's hospitals is a significant issue that should be addressed.<br>❑ Strongly Disagree<br>❑ Disagree<br>❑ Neutral	I believe unnecessary post-operative antibiotic use in children's hospitals is a significant issue that should be addressed.<br>❑ Strongly Disagree<br>❑ Disagree<br>❑ Neutral

	❑ Agree<br>❑ Strongly Agree	❑ Agree<br>❑ Strongly Agree
**15.**	I felt confident that facilitation skills could be effective in changing hospital practices related to post-operative antibiotic use<br>❑ Strongly Disagree<br>❑ Disagree<br>❑ Neutral<br>❑ Agree<br>❑ Strongly Agree	I feel confident that facilitation skills can be effective in changing hospital practices related to post-operative antibiotic use<br>❑ Strongly Disagree<br>❑ Disagree<br>❑ Neutral<br>❑ Agree<br>❑ Strongly Agree
**16.**	I believed that team-based approaches are critical to the successful de-implementation of unnecessary post-operative antibiotics<br>❑ Strongly Disagree<br>❑ Disagree<br>❑ Neutral<br>❑ Agree<br>❑ Strongly Agree	I believe that team-based approaches are critical to the successful de-implementation of unnecessary post-operative antibiotics<br>❑ Strongly Disagree<br>❑ Disagree<br>❑ Neutral<br>❑ Agree<br>❑ Strongly Agree
**Prac-tices**	**Before the workshop**	**After the workshop**
**17.**	I reviewed and used hospital-level data more actively to monitor post-operative antibiotic prescribing and patient outcomes.<br>❑ Strongly Disagree<br>❑ Disagree<br>❑ Neutral<br>❑ Agree<br>❑ Strongly Agree	I intend to review and use hospital-level data more actively to monitor post-operative antibiotic prescribing and patient outcomes.<br>❑ Strongly Disagree<br>❑ Disagree<br>❑ Neutral<br>❑ Agree<br>❑ Strongly Agree
**18.**	I used facilitation skills to enhance communication and team development within my hospital's antimicrobial stewardship program<br>❑ Strongly Disagree<br>❑ Disagree<br>❑ Neutral<br>❑ Agree<br>❑ Strongly Agree	I intend to use facilitation skills to enhance communication and team development within my hospital's antimicrobial stewardship program<br>❑ Strongly Disagree<br>❑ Disagree<br>❑ Neutral<br>❑ Agree<br>❑ Strongly Agree
**19.**	I actively participated in discussions or initiatives aimed at reducing unnecessary post-operative antibiotic use<br>❑ Strongly Disagree<br>❑ Disagree<br>❑ Neutral<br>❑ Agree	I intend to participate in discussions or initiatives aimed at reducing unnecessary post-operative antibiotic use<br>❑ Strongly Disagree<br>❑ Disagree<br>❑ Neutral<br>❑ Agree

	Strongly Agree	❑ Strongly Agree

Section 3. Open Ended Questions.
This final section invites you to share your thoughts about the workshop in your own words. Your feedback will help us understand your learning experience and improve future sessions.

**20.**	What were the most important thing(s) you learned?	________________________ ________________________
**21.**	What else would you add to the workshop?	________________________ ________________________
**22.**	What would you change about the workshop (time, structure, content)?	________________________ ________________________
**23.**	Do you have any other comments or thoughts?	________________________ ________________________
