# Supplementary material for: Dissemination of a facilitation strategy to de-implement unnecessary post-operative antibiotics at children's hospitals: The Optimizing Perioperative Antibiotic in Children (OPerAtiC) trial 2.0
Source: Implement Sci. 2025 Nov 10;20:49. doi: 10.1186/s13012-025-01460-5 (PMC12599022; doi:10.1186/s13012-025-01460-5)
Supplement: Supplementary file 2 — Supplementary Material 2 [file 13012_2025_1460_MOESM2_ESM.pdf]

ORDER SET CHANGE QUESTIONS

Interviewer: My name is _____, and I work for Washington University in St. Louis. Today I would like to take a few minutes to ask about what your antimicrobial stewardship team has done in in the past six (6) month regarding postoperative antibiotic prescribing with your surgical teams. Please feel free to stop me and let me know at any time if there is anything that you don't completely understand or if you have any questions

1.	Have you done anything to initiate or continue the process of changing order sets in the past six (6) month?	❑ yes ❑ No
2.	Have you identified order sets to change?	❑ yes ❑ No<br>If yes, how many? (numerical 1-5)
**If yes, ask the following questions for each order set identified.**		
2a.	Surgery/Procedure name:	______________________________
2b.	Were post-op antibiotics on them?	❑ yes ❑ No If yes, which one(s)?
2c.	What duration	❑ No antibiotics<br>❑ 24 hours<br>❑ 48 hours<br>❑ >/= 72 hours
2d.	Did you meet with the surgical team impacted by order sets?	❑ yes ❑ No If yes, which one(s)?
2e.	Have you changed the duration of any post op antibiotics from these order sets?	❑ removed ❑ shortened<br>❑ remained the same
	If removed or shortened, which antibiotics?<br><br>If removed or shortened, what was the date of change completion?<br><br>If shortened, to what duration?	_________________________________<br><br>_________________________________<br><br>_________________________________
3.	Have you helped initiate any new surgical order sets that do not have post operative antibiotics included?	❑ yes ❑ No If yes, which one(s)?
3a.	Surgery/Procedure name?	
4.	Other than order set changes, what else have you done this month to eliminate unnecessary post-operative antibiotic use?	
5.	Has anything happened (positive or negative) that would affect your ability	

	to eliminate unnecessary post-operative antibiotics?	
6.	Is there anything else you feel like we need to know regarding post-operative antibiotic use at your hospital in the past month?	❑ yes ❑ No If yes, please describe

Site name: ______________________________ Person providing answers: ______________________________

Completed by: ______________________ Date completed: ______________________________
